# From Puzzle to Progress: How Engaging With Neurodiversity Can Improve Cognitive Science

**DOI:** 10.1111/cogs.13255

**Published:** 2023-02-01

**Authors:** Marie A. R. Manalili, Amy Pearson, Justin Sulik, Louise Creechan, Mahmoud Elsherif, Inika Murkumbi, Flavio Azevedo, Kathryn L. Bonnen, Judy S. Kim, Konrad Kording, Julie J. Lee, Manifold Obscura, Steven K. Kapp, Jan P. Röer, Talia Morstead

**Affiliations:** a Faculty of Education & Society, University College London; b School of Health & Psychological Sciences, City, University of London; c School of Psychology, University of Sunderland; d Cognition, Values & Behavior, Ludwig Maximilian University of Munich; e Department of English Studies and Institute for Medical Humanities, Durham University; f Department of Psychology, University of Birmingham; g Department of Social Anthropology, University of Cambridge; h Department of Psychology, University of Cambridge; i School of Optometry, Indiana University Bloomington; j University Center for Human Values, Princeton University; k Departments of Neuroscience and Bioengineering, University of Pennsylvania and CIFAR; l Department of Psychology, New York University; m Independent Scholar; n Department of Psychology, University of Portsmouth; o Department for Psychology and Psychotherapy, Witten/Herdecke University; p Department of Psychology, University of British Columbia

**Keywords:** Cognitive diversity, Neurodiversity, Norms, Social cognition, Epistemic injustice, Scientific discovery

## Abstract

In cognitive science, there is a tacit norm that phenomena such as cultural variation or synaesthesia are worthy examples of cognitive diversity that contribute to a better understanding of cognition, but that other forms of cognitive diversity (e.g., autism, attention deficit hyperactivity disorder/ADHD, and dyslexia) are primarily interesting only as examples of deficit, dysfunction, or impairment. This status quo is dehumanizing and holds back much-needed research. In contrast, the neurodiversity paradigm argues that such experiences are not necessarily deficits but rather are natural reflections of biodiversity. Here, we propose that neurodiversity is an important topic for future research in cognitive science. We discuss why cognitive science has thus far failed to engage with neurodiversity, why this gap presents both ethical and scientific challenges for the field, and, crucially, why cognitive science will produce better theories of human cognition if the field engages with neurodiversity in the same way that it values other forms of cognitive diversity. Doing so will not only empower marginalized researchers but will also present an opportunity for cognitive science to benefit from the unique contributions of neurodivergent researchers and communities.

Cognitive science recognises the importance of diversity (Barrett, 2020; Bender, 2019; Henrich, Heine, & Norenzayan, 2010; Prather et al., 2022; Puthillam et al., 2022; Whitaker & Guest, 2020). It is puzzling, then, that neurodiversity has been absent from the discussion. After describing potential causes of this exclusion, we discuss its ethical and scientific consequences and propose how cognitive science can break new ground by embracing neurodiversity.

## 1 What is neurodiversity?

Neurodiversity^1^* refers to variation in human cognitive functioning (Walker, 2012, 2021), including takiwātanga*/autism*, ADHD*, dyslexia*, dyspraxia*, stuttering*, and plurality*. The neurodiversity paradigm* extends this view by recasting these experiences as differences (neurodivergence*) from common (neurotypical*) forms of cognitive functioning rather than as pathologized disorders (Asasumasu, 2015; Chapman, 2021; Dwyer, 2022; Elsherif et al., 2022; Walker, 2021). It provides an inclusive view of cognitive diversity, consolidating neurobiological differences with evolving sociocultural contexts of human experiences (Manalili, 2021).

The neurodiversity movement* applies this paradigm to political goals (e.g., resisting ableism* or promoting bodily autonomy; Arnold, 2017; Campbell, Constantino, & Simpson, 2019; Constantino, 2018; Walker, 2021). Here, however, we treat neurodiversity as a theoretical framework that makes heterogeneity a fundamental feature of processes such as executive function, attention, social cognition, communication, decision making, and motor control. As these are core topics in cognitive science, it is surprising that cognitive science has a scant engagement with the neurodiversity paradigm.

## 2 Why has cognitive science had poor engagement with neurodiversity?

Historical approaches misconceptualized neurodivergence as inherently disordered while framing neurotypical functioning as normative rather than common (Bertilsdottir-Rosqvist et al., 2022). This treated neurodivergence as scientifically interesting only when compared to assumed norms (Bertilsdottir-Rosqvist et al., 2022; Karmiloff-Smith, 2009) and cognitive theories of neurodivergence highlighted mechanisms of ‘impairment’ rather than manifestations of natural variation.

Some strands of cognitive science rest on similarly normative assumptions^2^: that research aims to discover rules or principles governing how the mind works, sometimes with the stronger assumption that these are optimal or rational. Such thinking may have evolutionary motivations, viewing biology as generating solutions to ecological problems that approximate optimal behavior (Körding, 2007; Ritter, Tehranchi, & Oury, 2019). Alternatively, it may derive from common frameworks, such as Bayesian modeling, which invoke rationality as a norm (Griffiths & Tenenbaum, 2006).

A normative frame commonly treats cognitive differences as variation on species-typical rules and principles due to culture or individual experience (Barrett, 2020) or as disordered breakdowns of those principles. However, neurodivergence is neither like cultural variation^3^ nor inherently disordered. Rather, it is a manifestation of biodiversity (Chapman, 2021).

When cognitive science frames neurodivergence as inherently disordered, it uses obsolete models of disability*. It would be unfortunate if this were the only frame of reference for areas of cognitive science less directly concerned with neurodiversity. Meanwhile, the humanities and social sciences have been applying the neurodiversity paradigm by rejecting oppressive medical models of disability in favor of social models*, which locate disability in the relationship between a person and their environment rather than being inherent to the person alone (Betts et al., in press; Chapman & Carel, 2022; Creechan, 2022; Kapp, 2013; Milton, 2012; Oliver, 1983, 2013; Stenning & Rosqvist, 2021).

## 3 Why is it wrong that cognitive science has not taken neurodiversity seriously?

It is unethical. Framing neurodivergence as “abnormal” is grounded in eugenics (Czech, 2018; Evans, 2014; Rutherford, 2022). Research maintaining this tradition dehumanizes neurodivergent people by treating them as objects—rather than agentive subjects—of research (Botha, 2021; Botha & Cage, 2022; Kapp, 2019). Theories built on deficit models are used to justify interventions that harm neurodivergent people (Yergeau, 2013), much like how conversion therapy harms gay and trans people by trying to “cure” them. Cognitive science should help prevent this by developing better theories of neurodivergence.

It is unjust. Conducting research about a marginalized group without their inclusion is epistemic injustice* (Byskov, 2021; Fricker, 2007). Neurodivergent people regularly face epistemic injustice (Catala, Faucher, & Poirier, 2021; Chapman & Carel, 2022). For example, even when tasks are modified to allow for cultural differences, similar calls for accommodation of neurodivergence are ignored (Hillary, 2020). Cognitive science should avoid injustice by empowering neurodivergent standpoints in research.

It is unscientific. For instance, although the theory of mind (ToM) is increasingly understood as comprising heterogeneous processes (Ahmed & Miller, 2011; Schaafsma, Pfaff, Spunt, & Adolphs, 2015; Warnell & Redcay, 2019), deficit-based accounts of neurodivergence talk in monolithic terms (e.g., describing autistic people as simply having a weaker ToM; Baron-Cohen, 2000). Claims about such “deficits” do not replicate reliably (Gernsbacher & Yergeau, 2019), further supporting the view that neurodiversity is more about differences than deficits (Astle & Fletcher-Watson, 2020). This view makes better sense of findings that communication failures are as much the fault of neurotypical people as they are of neurodivergent people (the double empathy problem*; Milton, 2012) as neurodivergent people understand each other even when neurotypicals do not (Crompton, Ropar, Evans-Williams, Flynn, & Fletcher-Watson, 2020). Cognitive science must develop more robust accounts of cognitive diversity that situate it in social contexts (e.g., second-person approaches; Schilbach et al., 2013).

## 4 Why will cognitive science be better if it seriously engages with neurodiversity?

Cognitive science can develop more accurate theories of cognitive diversity by working alongside neurodivergent communities, whereas sidelining of qualitative data from such communities has provided a false sense of objectivity. For instance, community-driven research on autism prioritizes different dimensions than research originating from neurotypical researchers. This has fed into newer quantitative studies and scale development (Garau et al., 2022; Wilson, 2022), though it is still in the minority. Going forward, such work should prioritize intersectionality (e.g., to combat Whiteness-centered stereotypes of autism; Botha, Dibb, & Frost, 2022). By analogy, trans healthcare has improved—not by doing more research into harmful conversion therapy or outdated conceptions of autogynephilia—but rather by doing different research that incorporates diverse trans perspectives (Serano, 2020).

Cognitive science can develop more general theories of cognitive diversity by broadening the range of diversity it considers worthy of attention. For instance, it recognizes the value of synaesthesia* in understanding cognitive diversity (Ward, 2019); neurodiversity should be no different. Kidd, Donnelly, and Christiansen (2018) highlight individual differences in language processing, yet limit their inquiry to neurotypicals. Future research should include non-deficit-framed language processing in neurodivergent people, including common interactions with attention or motor skills.

A weak way to include neurodiversity is in modeling previously unexplained variance (as might otherwise have been relegated to the random effects of a regression). This is weak because it is business as usual. It is like including autism status as a predictor to conclude that some people display worse ToM than others. It draws focus to how people vary along certain dimensions (such as ToM task scores) where neurotypicals are more central in the distribution and others more peripheral. This misconstrues neurotypicality as having an “average brain”^4^ and treats neurodivergence as defined by its deviation from that average. In any case, as many neurodivergent people do not have formal diagnoses, samples assumed to be neurotypical might not be.

There is a more radical way to embrace the message that neurodiversity is about difference, not deficit. It is analogous to the distinction in diversity metrics between “separation” (e.g., models of means and standard deviations) and “variety” (models of how different types are distributed in a group; Klein & Harrison, 2007). Whereas the weak strategy above treats diversity as separation, neurodiversity includes cognitive variety. This defocuses outcomes (e.g., variation in ToM performance) and instead highlights variety in cognitive mechanisms (e.g., heterogenous ways people tackle ToM tasks, whereby neurotypicality is “common” rather than “average”).^5^

In some areas, there is already a move in this direction. New research on brain-phenotype relations casts doubt on the validity of unitary cognitive models (Greene et al., 2022); in 3D perception, individuals vary in how they integrate different kinds of cues (Fulvio, Ji, & Rokers, 2021); a variety of mechanisms underlie ganglia circuit rhythms (Marder, Goeritz, & Otopalik, 2015); and some people engage linguistic processing when solving spatial problems, while others do not (Nedergaard, Wallentin, & Lupyan, 2022).

Converging on the point about “variety” from another angle: Even when there are evolutionary pressures on cognition and behavior, these are arguably pressures on the group rather than each individual. If there is no drive to optimize individual behavior, there is no single right way to learn, explore, manage attention, or make inferences. When intelligence is framed as a property of collectives (Falandays et al., 2022), true variety in cognitive processing and problem-solving—as endorsed by the neurodiversity paradigm—can contribute to group outcomes.

Neurodiversity thus presents an opportunity for cognitive science to build fundamentally new models of cognition. These could involve replacing single-agent reinforcement learning models with populations pursuing heterogenous strategies (related to neurodivergence by Dubois & Hauser, 2022; Yechiam, Arshavsky, Shamay-Tsoory, Yaniv, & Aharon, 2010). They could challenge the concept “theory of mind” (Williams, 2004), extending findings that ToM is not monolithic (Ahmed & Miller, 2011; Schaafsma et al., 2015; Warnell & Redcay, 2019) by showing how different neurotypes use different strategies in doing ToM tasks. Models relating group-level success to individual diversity could shift away from varying continuous parameters (Sulik, Bahrami, & Deroy, 2021a) toward studying distinct strategies (Devezer, Nardin, Baumgaertner, & Buzbas, 2019).

Finally, there are benefits for cognitive science *qua* science. The above points about ethics, justice, and science converge in the larger question—from Indigenous and feminist philosophy of science (Harding & Hintikka, 2003; Pownall et al., 2021; Smith, 2021)—of who gets to contribute to knowledge. A marginalized standpoint allows individuals to see what those in positions of privilege cannot, yielding unique epistemic advantages (Friesen & Goldstein, in press). Diversity is especially important for scientific discovery: identifying questions, framing problems, or uncovering insights (Sulik, Bahrami, & Deroy, 2021b). Neurodivergent people explore in different ways than neurotypicals (Dubois & Hauser, 2022; Yechiam et al., 2010), and exploration is part of discovery (Devezer et al., 2019). A variety of strategies—incorporating research led by both neurodivergent and neurotypical people—could lead to better long-term advances. Increased promotion of Open Science/Scholarship aims may also improve inclusion of neurodivergent researchers (Azevedo et al., 2022; Elsherif et al., 2022).

Without neurodiversity, cognitive science offers an impoverished account of cognitive diversity. It dehumanizes neurodivergent communities, and it is missing out on the invaluable contributions of neurodivergent researchers.

## 5 Recommendations

For cognitive science as an institution:

1. Explicitly include neurodiversity in institutional diversity statements.
2. Reject models or theories that frame neurodivergence as inherently disordered.

For research comparing neurodivergent and neurotypical populations:

1. Critically test assumptions that neurodivergence necessarily involves deficits.
2. Promote neurodivergent-led research and community involvement.
3. Evaluate whether common tasks are fair measures of heterogeneous cognitive processes.

For research on topics commonly implicated in neurodivergence (e.g., executive function, ToM, decision making):

1. Do not assume that samples are (or should be) neurotypical.
2. Explore whether human cognition offers a variety of ways to approach superficially similar tasks.

## Positionality statement

The meaning of neurodiversity may differ among authors of this article due to our various lived experiences, neurodivergence, ethnicity, gender, epistemological, ontological, and/or methodological perspectives. However, we are united in combating ableism and in believing that cognitive science cannot be truly representative until it empowers and engages with neurodivergent populations and in emphasizing the intersectional nature of neurodiversity. Social and institutional barriers—such as higher rates of misdiagnosis/misidentification of neurodivergence—disproportionately impact disabled people, people of color, and women (Davis, Solomon, & Belcher, 2022; Diemer, Gerstein, & Regester, 2022; Jack, 2011; Malone et al., 2022; Zener, 2019). Neurodivergence also intersects with queerness and variant gender identity (Walker, 2021). Some forms of neurodivergence are more stigmatized than others (Manalili, 2021).

## Notes

1 Terms marked * are explained in the accompanying glossary (https://osf.io/hujzq). When the terms are multi-word phrases, they are marked in italics.

2 Cognitive science is not monolithic in its assumptions, though it is all the more puzzling that other strands of research—falling under the broad umbrella of cognitive science but not motivated by such normative assumptions—have ignored neurodiversity.

3 Though degrees and forms of acceptance of neurodiversity vary across cultures

4 Whatever *that* may mean.

5 Richters (2021) makes the stronger claim that an outcome-variable oriented (in our terms, “separation”) approach to individual differences assumes homogeneity of underlying psychological structures and processes. For a recent account of philosophical characterizations of cognitive diversity and how they map onto statistical models, see Ward (2022).
